# Longitudinal Analysis of Mitochondrial D-Loop Methylation and Copy Number in Peripheral Blood: Epigenetic Signatures of Alzheimer’s Disease Progression and Aging

**DOI:** 10.3390/ijms27031477

**Published:** 2026-02-02

**Authors:** Bartolo Rizzo, Michele Rossi, Riccardo Rocco Ferrari, Elisa Pellegrini, Francesca Dragoni, Rosalinda Di Gerlando, Evelyne Minucchi, Antonio Guaita, Tino Emanuele Poloni, Stella Gagliardi, Annalisa Davin

**Affiliations:** 1 Golgi Cenci Foundation, 20081 Abbiategrasso, Italy; b.rizzo@golgicenci.it (B.R.); m.rossi@golgicenci.it (M.R.); r.ferrari@golgicenci.it (R.R.F.); e.pellegrini@golgicenci.it (E.P.); a.guaita@golgicenci.it (A.G.); e.poloni@golgicenci.it (T.E.P.); a.davin@golgicenci.it (A.D.); 2 IRCCS Mondino Foundation, 27100 Pavia, Italy; francesca.dragoni@mondino.it (F.D.); rosalinda.digerlando@mondino.it (R.D.G.); evelyne.minucchi@mondino.it (E.M.)

**Keywords:** longitudinal study, methylation, mitochondria, Alzheimer’s disease, pyrosequencing, aging, epigenetic signature, dementia, cognitive decline, mild cognitive impairment

## Abstract

Alzheimer’s disease (AD), the leading cause of dementia, is expected to markedly increase in prevalence in the coming decades. Beyond amyloid and tau pathologies, accumulating evidence suggests that mitochondrial dysfunction and impaired protein homeostasis play crucial roles in AD onset and progression. Building on our previous identification of molecular signatures associated with disease progression, this study investigated whether epigenetic alterations of mitochondrial DNA (mtDNA) contribute to cognitive decline. Specifically, we analyzed the methylation status of the mtDNA regulatory D-loop region and mtDNA copy number in blood-derived DNA samples from 75 participants who we followed longitudinally over eight years. Subjects were classified into four groups according to clinical progression from healthy cognition to mild cognitive impairment (MCI) and AD. Using a linear mixed-effects model, we observed significant differences in methylation dynamics and mtDNA copy number across groups and time points. Healthy controls showed a progressive increase in D-loop methylation, whereas individuals converting to AD exhibited a marked decrease in its level. An opposite trend was evidenced for mtDNA copy number. These findings suggest that reduced D-loop methylation and increased mtDNA are associated with mitochondrial dysfunction and disease progression, whereas increased methylation may represent a possible protective mechanism.

## 1. Introduction

AD is a neurodegenerative disorder and is currently recognized as the leading cause of dementia worldwide [1]. Advanced age represents a particularly relevant risk factor in these sporadic cases, as both prevalence and incidence increase sharply in older populations. This observation suggests that biological mechanisms underlying the aging process may actively contribute to the neurodegenerative features typical of AD [2]. Among genetic risk factors for sporadic Alzheimer’s disease, the apolipoprotein E (*APOE*) ε4 allele represents the strongest susceptibility variant, influencing both disease risk and age at onset. In addition, polymorphisms in the neighboring *TOMM40* gene, including the rs10524523 poly-T repeat and rs2075650, have been investigated for their potential role in modulating disease susceptibility and progression, either independently or in interaction with *APOE* genotypes [3,4,5].

Neurodegeneration in AD, including synaptic failure and neuronal loss, has been attributed to the accumulation of extracellular toxic β-amyloid (Aβ) oligomers, protein aggregates, and intracellular neurofibrillary tangles (NFTs) composed of hyperphosphorylated tau (pTau), causing synaptic and mitochondrial dysfunction and region-specific atrophy with alterations in cerebral glucose metabolism and mitochondrial dysfunction [6]. From a pathological standpoint, AD is characterized by extracellular senile plaques formed by Aβ peptides, NFTs containing pTau, cerebral amyloid angiopathy due to Aβ deposition on vessel walls, and widespread neuronal loss. Consequently, reducing Aβ production and/or enhancing its clearance along with that of pTau remains a leading therapeutic strategy [7].

Several hypotheses, alternative to the “amyloid cascade hypothesis”, have been proposed to explain the etiology of AD, ranging from the “mitochondrial cascade hypothesis” [8] to chronic neuroinflammation [9] and impaired proteostasis, which is also tightly linked to synaptic failure [10]. A growing body of evidence indicates that AD is a highly complex and heterogeneous disorder, necessitating innovative methodological approaches to better understand its intricacies. Within this framework, genetic and transcriptomic analyses have shown significant potential in uncovering the molecular mechanisms driving AD pathogenesis. Transcriptomic studies provide the opportunity to identify differentially expressed genes (DEGs) and alternative regulatory pathways that have been associated with neurodegenerative processes [11,12].

An emerging perspective proposes that age-related alterations such as mitochondrial dysfunction, oxidative stress, loss of protein homeostasis, and neuroinflammation play a critical role in the onset of age-related diseases, including AD [13]. Notably, mitochondrial dysfunction has been identified as one of the main pathological drivers in AD [14]. Although mitochondria are commonly referred to as the “powerhouses” of the cell, they are also involved in fundamental processes such as programmed cell death, immune responses, macromolecule biosynthesis, and calcium homeostasis. During aging, mitochondrial functionality declines progressively due to impaired oxidative phosphorylation (OXPHOS), increased oxidative stress mediated by reactive oxygen species (ROS), and defective mitochondrial recycling [15]. In AD, a reduction in glucose utilization is one of the hallmarks of mitochondrial alterations, indicating a failure of the energy production system [16]. Additional mitochondrial dysfunctions in AD include ROS overproduction, calcium imbalance, and abnormal mitochondrial dynamics [17].

mtDNA is also subject to epigenetic regulation. Mechanisms such as DNA methylation, chromatin remodeling, and histone post-translational modifications regulate various aspects of mitochondrial function, inflammatory responses, and cell development [18]. In this context, the term “mitoepigenetics” has been introduced to describe epigenetic processes that regulate mtDNA transcription and replication [19]. While mtDNA typically exhibits lower levels of methylation and hydroxymethylation than nuclear DNA (nDNA), these modifications are dynamic and play a key role in controlling mtDNA expression [20]. The D-loop regulatory region is among the most methylated regions of the mitochondrial genome, though average methylation levels typically remain below or around 5% [20,21,22]. The D-loop, a noncoding region of approximately 1.1 kb containing key regulatory elements for mtDNA transcription and replication, is particularly vulnerable to such epigenetic modifications [23]. Alterations in D-loop methylation may affect mtDNA stability and transcriptional activity, thereby potentially contributing to changes in mtDNA copy number observed in AD patients. Measurement of mtDNA copy number thus provides an additional layer to assess mitochondrial function and its dysregulation in neurodegeneration.

In our recent work [12], we observed that several genes associated with mitochondrial dysfunction were significantly altered in AD. RNA-sequencing analysis revealed a greater number of deregulated genes in brain regions more severely affected by the disease. Among these, two molecular targets hemoglobin (*HBB*) and insulin growth factor 2 (*IGF2*) were consistently dysregulated across all analyzed brain regions.

Based on these findings and corroborating literature, we hypothesized that mitochondrial dysfunction plays a central role in AD pathogenesis. We thus investigate the potential involvement of epigenetic alterations in mtDNA, particularly in the D-loop region, and mtDNA copy number changes. These alterations suggest a potential association that, if confirmed in larger cohorts controlling for hematological confounders, might offer preliminary value for monitoring disease progression. This line of research could eventually support the long-term goal of developing personalized medicine strategies. Recent studies have examined the overlap between aging markers and AD, suggesting that the pathogenesis of AD could reflect an intensified or accelerated form of brain aging, with mitochondrial dysfunction emerging as a crucial focal point in this convergence [2].

In recent years, increasing attention has been directed toward identifying the earliest preclinical stages of AD. There is a growing consensus that the pathophysiological processes of AD begin many years before the onset of clinically overt symptoms, thus supporting the concept of a pre-symptomatic or preclinical phase of the disease [24].

In order to test these hypotheses, we conducted a retrospective longitudinal study aimed at investigating a possible role in the pre-symptomatic and progressive phases of dementia, as well as in healthy aging. The study investigated D-loop methylation and mtDNA copy number in blood samples of subjects with four different evolutionary profiles over time: those who remain healthy, those who evolve to AD and those who convert from MCI to AD.

## 2. Results

This study investigated D-loop methylation and mtDNA gene copy number in the blood sample in the prodromic phase of AD. Results were obtained from a longitudinal study conducted on 75 patients, analyzing methylation levels of the D-loop region of mitochondrial DNA at three different time points: T0, T1 after 4 years, and T2 after another 4 years. Subjects were divided into four groups based on the progression of the cognitive decline over time: Group 0 (HC-HC-HC, n = 34): patients with no cognitive deficits at any of the three time points, serving as healthy controls (HC). Group 1 (HC-HC-AD, n = 12): patients who were cognitively unimpaired at the first and second time points but received a diagnosis of AD at T2. Group 2 (HC-MCI-AD, n = 20): patients who were cognitively unimpaired at T0, then defined mild cognitive impairment (MCI) at T1, and diagnosed with AD at T2. Group 3 (MCI-MCI-AD, n = 9): patients with MCI at both T0 and T1 and diagnosed with AD at T2.

The analyses were conducted on whole-blood DNA. Because both mtDNA copy number and mitochondrial DNA methylation can be influenced by blood cell composition, we assessed whether white blood cell and platelet counts differed between groups or over time. We found no significant differences in these counts across groups or time points, and repeated-measures analyses revealed no significant correlations between D-loop methylation or mtDNA copy number and blood cell counts.

The results obtained show significant differences both between groups and between time points within each group.

### 2.1. Analysis of D-Loop Methylation Profile in Longitudinal Study

Table 1 summarizes the results of the linear mixed-effects model used to evaluate longitudinal changes in D-loop methylation among the different conversion groups. The model included time, group, and their interaction as fixed effects, and was adjusted for age, sex, TOMM40 genotype, and APOE genotype. This analysis shows a decrease in D-loop methylation in AD patients compared with HC subjects. In particular, at T0, Group 2 (HC–MCI–AD) showed significantly higher D-loop methylation levels compared with Group 0 (HC–HC–HC) *p* = 0.004). At T2, a significant reduction in D-loop methylation was observed in both Group 2 (HC-MCI-AD) (*p* < 0.001) and Group 1 (HC-HC-AD) (*p* = 0.001) relative to Group 0 (HC-HC-HC) at baseline. The remaining values are reported in Table 1.

**Figure 1:**
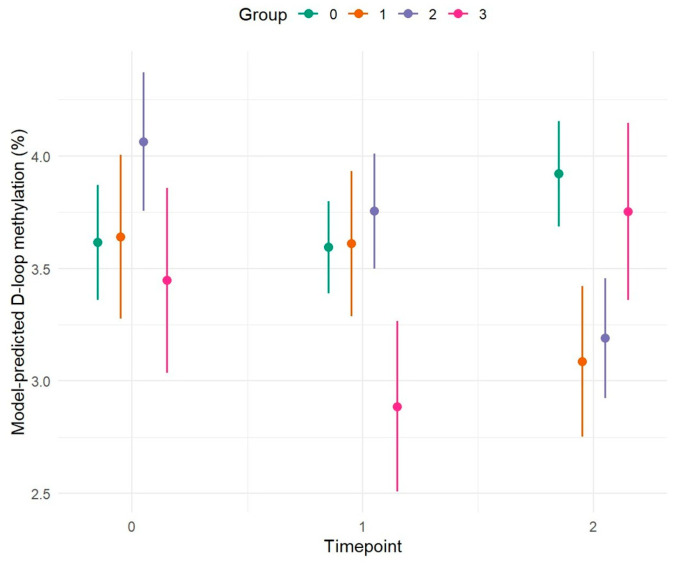
Longitudinal trend of D-loop methylation levels as a function of time, assessed at baseline (T0), after four years (T1), and after an additional four years (T2), across the different study groups. Summary of study cohort: Group 0 (HC-HC-HC, n = 34) (green) = Patients with no cognitive deficits at any time point (healthy controls); Group 1 (HC-HC-AD, n = 12) (orange) = Patients cognitively healthy at two time points, diagnosed with AD at T2; Group 2 (HC-MCI-AD, n = 20) (blue) = Patients cognitively healthy at T0, with MCI at T1, and AD at T2; Group 3 (MCI-MCI-AD, n = 9) (fuchsia) = Patients with MCI at both T0 and T1, and AD at T2. Lines represent marginal predictions estimated from a linear mixed-effects model with a random intercept for subject, adjusted for age and sex. Shaded areas indicate 95% confidence intervals.

Figure 1 highlights a different methylation pattern between Group 0 and Groups 1, 2, and 3. Specifically, Group 0 (HC-HC-HC) shows a constant increase in methylation levels across the different time points between T0 and T2. Conversely, Groups 1 (HC-HC-AD) and Group 2 (HC-MCI-AD), diagnosed with AD at T2, show an opposite trend, with a reduction in methylation at T2. The results of the proposed longitudinal study show an association between D-loop methylation levels and the progression of cognitive decline in the subjects analyzed. Specifically, Group 0 (HC-HC-HC), where subjects do not undergo pathological evolution, shows a continuous increase in methylation over time, suggesting a possible protective or compensatory role of this epigenetic phenomenon in maintaining cognitive health. Conversely, Groups 1 (HC-HC-AD) and Group 2 (HC-MCI-AD), diagnosed with AD at time T2, show a significant drop in methylation, especially at the time of clinical conversion. This opposite trend, supported by statistically significant *p*-values, suggests that a decrease in D-loop methylation may be associated with the onset and progression of the disease. Furthermore, the inclusion of the previously described variables did not result in any difference. The exception is sex, which appears to have a marginal effect (*p* = 0.056) (Table 1).

#### 2.1.1. Comparison of D-Loop Methylation Levels Between Groups at Individual Time Points

To analyze the evolution of cognitive trajectories across the four groups (Group 0 –Group 3), pairwise comparisons were performed at each time point (T = 0, 1, 2) (Figure 1). At the T0 of the study, significant differences were observed between Group 0 (HC-HC-HC) and Group 2 (HC-MCI-AD) (*p* = 0.022) and between Group 2 (HC-MCI-AD) and Group 3 (MCI-MCI-AD) (*p* = 0.029). These results suggest that Group 2 (HC-MCI-AD) already exhibits distinct baseline characteristics, differentiating it from both cognitively healthy subjects and those with MCI. It is plausible that this group represents an early transitional phase, characterized by subclinical or pre-symptomatic alterations that preceded the clinical manifestation of MCI. Other comparisons did not show statistically significant differences (*p* > 0.05), indicating substantial balance among the remaining groups at this initial stage.

After the T1, differences between groups became more pronounced. Group 3 (MCI-MCI-AD) was significantly different from Group 0 (HC-HC-HC) (*p* = 0.003), Group 1 (HC-HC-AD) (*p* = 0.016), and Group 2 (HC-MCI-AD) (*p* ≤ 0.001). This pattern suggests that Group 3 (MCI-MCI-AD) follows a more rapid or pronounced cognitive decline trajectory, consistent with its classification as MCI already at T0 and T1.

At T2, the most relevant differences were observed between Group 0 (HC-HC-HC) vs. Group 1 (HC-HC-AD) (*p* ≤ 0.001), Group 0 (HC-HC-HC) vs. Group 2 (HC-MCI-AD) (*p* < 0.0001), and Group 1 (HC-HC-AD) vs. Group 3 (MCI-MCI-AD) (*p* = 0.031). The comparison between Group 2 (HC-MCI-AD) and Group 3 (MCI-MCI-AD) showed a trend toward significance (*p* = 0.056) but did not reach the conventional threshold. These results indicate that over time, distinctions between groups progressively increase while minor differences emerge in the early stages between at-risk subjects and controls, in the final stage differences are more pronounced between AD patients and HC, suggesting a progressive convergence of cognitive profiles among the pathological groups (Figure 1 and Table 2).

#### 2.1.2. Comparison of Methylation Levels Across Different Time Points Within Each Group

To analyze the temporal evolution of methylation within each group, pairwise comparisons were performed between the three observational time points (T0, T1, T2), stratified by group (Group 0–Group 3) (Figure 1).

In Group 0 (HC-HC-HC), no statistically significant differences were observed between the different time points. Group 0 exhibits overall stability, with a slight upward trend in methylation over time, likely associated with physiological aging processes.

Similarly, in Group 1 (HC-HC-AD), no statistically significant differences were observed between time points T0-T1 (*p* = 0.989), T0-T2 (*p* = 0.085), and T1-T2 (*p* = 0.064).

In Group 2 (HC-MCI-AD), significant temporal differences emerged, with evident changes between observation points. The T0-T2 comparison was highly significant (*p* ≤ 0.001), and T1-T2 also showed a significant difference (*p* = 0.007), whereas T0-T1 did not reach significance (*p* = 0.219).

In Group 3 (MCI-MCI-AD), a significant difference was observed between T1 and T2 (*p* = 0.003), while T0-T1 (*p* = 0.090) and T0-T2 (*p* = 0.541) were not significant. Overall, the results show a heterogeneous temporal pattern across groups (Figure 1 and Table 3).

### 2.2. Analysis of mtDNA Copy Number in Longitudinal Study

Table 4 summarizes the results of the linear mixed-effects model used to evaluate longitudinal changes in mtDNA copy number among the different conversion groups. The model included time, group, and their interaction as fixed effects, and was adjusted for age, sex, TOMM40 genotype, and APOE genotype. The analysis revealed a progressive increase in mtDNA copy number over time in groups converting to AD compared with Group 0 (HC–HC–HC). Specifically, At T0, Group 1 (HC–HC–AD), Group 2 (HC–MCI–AD), and Group 3 (MCI–MCI–AD) showed significantly lower mtDNA copy number levels compared with Group 0 (HC–HC–HC) (all *p* ≤ 0.023). At T1, Group 1 exhibited a significant increase in mtDNA copy number relative to Group 0 at baseline (*p* = 0.006). At T2, Group 1 and Group 2 exhibited a significant increase in mtDNA copy number relative to Group 0 at baseline (both *p* < 0.001). Additional results are reported in Table 4.

Results reveal a different copy number pattern between Group 0 and Groups 1, 2, and 3.

**Figure 2:**
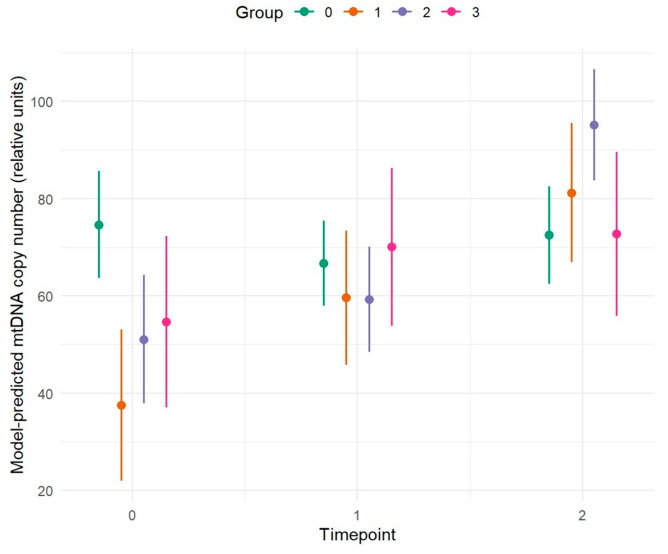
Longitudinal trend of mtDNA copy number levels as a function of time, assessed at baseline (T0), after four years (T1), and after an additional four years (T2), across the different study groups. Summary of study cohort: Group 0 (HC-HC-HC, n = 34) (green) = Patients with no cognitive deficits at any time point (healthy controls); Group 1 (HC-HC-AD, n = 12) (orange) = Patients cognitively healthy at two time points, diagnosed with AD at T2; Group 2 (HC-MCI-AD, n = 20) (blue) = Patients cognitively healthy at T0, with MCI at T1, and AD at T2; Group 3 (MCI-MCI-AD, n = 9) (fuchsia) = Patients with MCI at both T0 and T1, and AD at T2. Lines represent marginal predictions estimated from a linear mixed-effects model with a random intercept for subject, adjusted for age and sex. Shaded areas indicate 95% confidence intervals.

Group 0 (HC-HC-HC) shows a slight decrease in copy number levels over time, suggesting that aging in healthy subjects does not lead to significant alterations in mitochondrial function, thereby allowing maintenance of cognitive health. Conversely, Group 1 (HC-HC-AD), Group 2 (HC-MCI-AD), and Group 3 (MCI-MCI-AD) exhibit a significant increase in copy number, particularly coinciding with clinical conversion to an AD. Age, TOMM40 genotype, and APOE genotype were included as covariates, for which no significant differences were observed; however, sex appears to have a potential effect, with a *p*-value of 0.056 (Figure 2 and Table 4).

#### 2.2.1. Comparison of Copy Number Levels Between Groups at Individual Time Points

Pairwise comparisons were performed between the different groups (Group 0–Group 3) at each observation time point (T = 0, 1, 2) (Figure 2).

At T0, statistically significant differences emerged between Group 0 (HC-HC-HC) and Group 1 (HC-HC-AD) (*p* < 0.001) and between Group 0 (HC-HC-HC) and Group 2 (HC-MCI-AD) (*p* = 0.002). All other comparisons did not reach statistical significance (all *p* > 0.05). These results indicate that, at the start of the observation period, Group 0 (HC-HC-HC) differs markedly from Group 1 (HC-HC-AD) and Group 2 (HC-MCI-AD), while no relevant differences are observed among the other groups.

At T1, no significant differences were detected between the groups (all *p* > 0.05) suggesting substantial homogeneity among the conditions at this time point.

At the final time point, a significant difference was observed between Group 0 (HC-HC-HC) and Group 2 (HC-MCI-AD) (*p* = 0.003), while all other comparisons were not significant (*p* > 0.05). The comparison between Group 2 (HC-MCI-AD) and Group 3 (MCI-MCI-AD) showed a trend towards significance (*p* = 0.083) but did not exceed the conventional threshold. This pattern suggests that, by the end of the observation period, Group 2 (HC-MCI-AD) again diverges from Group 0 (HC-HC-HC), confirming a temporal pattern of divergence. Overall, the results indicate that differences between groups are not constant over time. (Figure 2 and Table 5)

#### 2.2.2. Comparison of Copy Number Levels Across Different Time Points Within Each Group

To evaluate the temporal trends of the analyzed variables, pairwise comparisons were conducted between observation time points (T0, T1, T2) within each group (Group 0–Group 3) (Figure 2).

For Group 0 (HC-HC-HC) no statistically significant differences were observed across time points indicating substantial stability of the measures over time.

In Group 1 (HC-HC-AD), temporal variations were observed. In particular, the T0–T2 comparison was highly significant and showed an increasing trend (*p* ≤ 0.001), while T0–T1 (*p* = 0.063) and T1–T2 (*p* = 0.072) showed trends toward significance, also in an increasing direction. These results suggest a progressive change from the initial to the final time point, with differences becoming more pronounced over the long term.

In Group 2 (HC-MCI-AD), copy number increased, with significant differences observed between T0 and T2 (*p* < 0.001) and between T1 and T2 (*p* < 0.001), while the T0–T1 comparison was not significant (*p* = 0.543). These data indicate marked changes, with a significant variation emerging at T2, suggesting a strong temporal dynamic in this group.

For Group 3 (MCI-MCI-AD), none of the comparisons between time points reached statistical significance (T0–T1: *p* = 0.352; T0–T2: *p* = 0.305; T1–T2: *p* = 0.969). Nonetheless, an increasing trend in mitochondrial DNA copy number is evident. Overall, the results show that temporal patterns vary among groups. (Figure 2 and Table 6).

### 2.3. Association Between MMSE Scores, DNA Methylation, and mtDNA Copy Number

To further investigate the molecular mechanisms associated with cognitive decline and progression toward AD, we examined the relationship between Mini-Mental State Examination (MMSE) scores and two longitudinally measured biological parameters: DNA methylation and mtDNA copy number. These analyses aimed to determine whether specific molecular alterations correspond to cognitive status during follow-up.

#### 2.3.1. Association Between MMSE and D-Loop DNA Methylation

**Figure 3:**
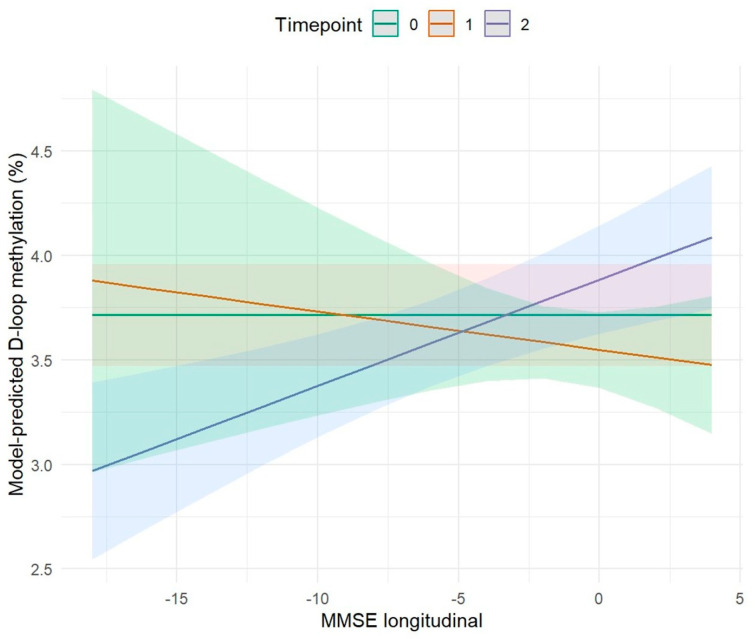
Effect of longitudinal MMSE on changes in D-loop methylation levels over time. Lines represent marginal predictions estimated from a linear mixed-effects model with a random intercept for subject, adjusted for age and sex. Shaded areas indicate 95% confidence intervals. The green line refers to time point 0, the orange line to time point 1, and the purple line to time point 2.

In this study, we evaluated the association between cognitive performance, measured by the Mini-Mental State Examination (MMSE), and DNA methylation levels at follow-up (Figure 3).

Our results revealed a statistically significant positive effect between MMSE longitudinal variation and DNA methylation at T2 (*p* ≤ 0.001). Increases in MMSE scores over time were associated with increased methylation levels, while decreases in cognitive performance corresponded to reduced methylation. This pattern is consistent with observations within our cohort: individuals who remained cognitively healthy exhibited higher levels of methylation compared with those who later converted to AD (Figure 3 and Table 7).

#### 2.3.2. Association Between MMSE and mtDNA Copy Number

**Figure 4:**
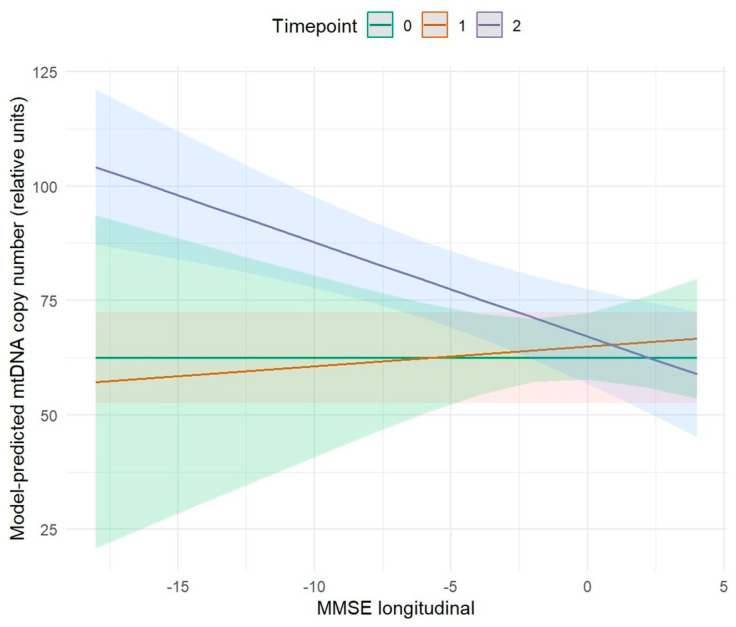
Effect of longitudinal MMSE on changes in mtDNA copy number levels over time. Lines represent marginal predictions estimated from a linear mixed-effects model with a random intercept for subject, adjusted for age and sex. Shaded areas indicate 95% confidence intervals. The green line refers to time point 0, the orange line to time point 1, and the purple line to time point 2.

We further investigated the relationship between MMSE performance and mtDNA copy number at T2 (Figure 4).

Our results revealed a statistically significant negative effect between MMSE longitudinal variation and mtDNA copy number at T2 (*p* ≤ 0.001). Increases in MMSE scores over time were associated with decreases number of mitochondrial genes copies, while decreases cognitive performance corresponded to increased mtDNA copy number. Within our study population, this relationship was reflected in the clinical trajectory of subjects: cognitively stable controls showed lower mtDNA copy numbers, whereas individuals who subsequently developed AD exhibited higher copy numbers. Overall, the observed association supports the hypothesis that alterations in mitochondrial dynamics accompany cognitive decline and may serve as early molecular indicators of progression toward AD (Figure 4 and Table 8).

## 3. Discussion

Recent research indicates that mitochondrial epigenetic alterations are associated with AD and may contribute to mitochondrial dysfunction early in the disease process. In fact, many studies have reported differences in methylation patterns of mtDNA in AD patients compared with controls, suggesting that mtDNA epigenetic regulation could influence gene expression, replication, and mitochondrial performance in neurodegeneration [25,26,27]. Various group investigations have reported an association between D-loop methylation levels and mtDNA copy number in diverse cell types, generally demonstrating an inverse correlation [20,23,28,29], supporting the hypothesis that D-loop methylation modulates mtDNA replication and mitochondrial gene expression. However, conflicting data have also been published, such as the positive correlation documented by Byun and colleagues [30].

Starting from these assumptions, the primary objective of this study was to investigate the alterations in mitochondrial D-loop methylation and mtDNA copy in AD. To achieve this, we quantified methylation levels of the regulatory D-loop region of mtDNA in peripheral blood within a longitudinal cohort of 75 patients evaluated at three distinct time points: T0, T1 (after 4 years), and T2 (after an additional 4 years). Participants were systematically grouped based on the evolution of their cognitive status and for each sample both mtDNA copy number and methylation levels of D-loop region have been studied.

Mitochondrial DNA copy number and D-loop methylation could potentially be influenced by peripheral blood cell-type composition. In our cohort white blood cell and platelet counts did not differ significantly between groups across the three time points. Furthermore, repeated-measures analyses showed no significant correlations between D-loop methylation or mtDNA copy number and these cell counts. These findings suggest that the longitudinal changes we observed are unlikely to be solely driven by shifts in peripheral blood cell composition, suggesting that our findings reflect disease-related mitochondrial epigenetic alterations rather than confounding hematological or immune changes.

### 3.1. Temporal Trajectories of mtDNA D-Loop Methylation: Mapping the Transition from Cognitive Health to Alzheimer’s Disease

Our data demonstrated dynamic differences in D-loop methylation closely linked to the progression of cognitive decline. In accordance with published data, in Group 1 (HC_HC_AD) and Group 2 (HC_MCI_AD), which converted to AD at T2, a significant reduction in methylation was observed, particularly conspicuous at the time of conversion. In contrast, Group 0 (HC-HC-HC) exhibited a steady or slightly increasing level of methylation, suggesting a potentially protective or physiologically consistent role. Prior literature has reported reduced mtDNA methylation levels in the entorhinal cortex of patients with early-stage AD pathology, and dynamic changes in mtDNA methylation have also been observed in APP/PSEN1 mouse models as the disease progresses, highlighting consistent alterations across human and experimental work [25]. Subsequently, an independent study reported diminished D-loop methylation levels in peripheral blood from AD patients relative to controls [26]. Moreover, findings by Stoccoro et al. (2017 and 2022) [26,31] showed that D-loop methylation, in peripheral blood, is sensitively modulated in the early phases of the disease. Our results confirming the literature, aligning with this evidence, suggest that peripheral D-loop methylation levels may reflect different stages of AD progression. Our results, supported by the mentioned published literature [26,27], strongly suggest that decreased D-loop methylation is associated with disease progression.

Our observations are further substantiated by animal model studies documenting decreased D-loop methylation, mtDNA copy number, and gene expression in the hippocampus of AD mice [32,33]. More specifically, our analyses indicated that at T0, Group 2 (HC-MCI-AD) displayed characteristics distinct from cognitively healthy subjects and those with MCI, implying that alterations in D-loop methylation may precede the clinical onset of cognitive decline. This pattern indicates that changes occur in a later stage of the observation period, when subjects, already classified as MCI at T0 and T1, progress toward full-blown AD. The initial stability followed by late variation may reflect a collapse of cerebral compensatory capacity or a progressive accumulation of molecular damage that exceeds a critical threshold only in the final phase. Overall, the results show a heterogeneous temporal pattern across groups. Group 0 (HC-HC-HC) exhibits overall stability, with a slight upward trend in methylation over time, likely associated with physiological aging processes. Group 1 (HC-HC-AD) shows a similar behavior in the first two time points, but a decrease in methylation levels at T2, coinciding with conversion to AD, suggesting epigenetic dysregulation associated with disease onset. Group 2 (HC-MCI-AD) demonstrates significant changes between T0-T2 and T1-T2, indicative of a dynamic and progressive process consistent with an active transitional phase toward the disease. Finally, Group 3 (MCI-MCI-AD) shows significant variation only between T1 and T2, indicating that epigenetic changes manifest at a more advanced stage of the disease. These findings suggest that epigenetic modifications, particularly methylation levels, follow different temporal trajectories in healthy subjects and those with cognitive decline. While the trend appears stable or physiological in controls, more pronounced and temporally concentrated changes are observed in groups progressing toward Alzheimer’s, potentially reflecting molecular processes associated with loss of neuronal plasticity, neuroinflammation [34,35], or impaired cerebral energy metabolism.

Blanch et al. [25] reported that D-loop methylation shifted during disease development in an AD mouse model, reinforcing the idea that methylation levels in this mitochondrial region are sensitive to distinct disease phases.

### 3.2. The Interplay Between mtDNA D-Loop Methylation and Copy Number: A Compensatory but Deleterious Mechanism in Alzheimer’s Progression

Our study examined how mtDNA copy number fluctuates over an 8-year period in peripheral blood of subjects who convert from cognitively healthy status to AD, while remaining consistent in controls who maintain cognitive health. In our data, mtDNA copy number was significantly reduced at T0 in patients who later developed AD compared with subjects who remained cognitively healthy. Over time, while copy number remained relatively stable in controls, it showed an upward tendency in subjects progressing to AD, suggesting a potential compensatory mechanism aimed at counteracting the increasing mitochondrial dysfunction but also providing a possible therapeutic target aimed at reducing the number of mtDNA and reducing oxidative stress state, as one of the main characteristics of the disease.

mtDNA is highly susceptible to oxidative damage due to its proximity to ROS production, lack of histones, and limited repair mechanisms. Excessive ROS, generated via electron leakage, drives the oxidative stress and biomolecular damage characteristically observed in AD [36,37,38,39,40,41]. Numerous investigations have reported a reduction in mtDNA copy numbers in older individuals. In a large cohort of Sardinian subjects, mtDNA levels in lymphocytes declined considerably with age [42]. More significant reductions were observed in peripheral blood starting at age 50 and even more drastically in older adults [43,44]. Notably, among individuals over 58 years old, a low mtDNA copy number was linked to elevated mortality, poorer health status, and reduced cognitive and physical function [44]. However, studies on long-lived families (nonagenarians and centenarians) have yielded contradictory results, reporting both lower and higher mtDNA levels compared with middle-aged individuals, implying a complex and non-linear relationship among mtDNA, aging, and longevity [45,46]. Deregulated mtDNA replication is commonly identified in both cerebral tissues and the peripheral blood of individuals with AD [47,48].

Previous studies have reported lower mtDNA levels in patients with MCI and AD, though an increase in mtDNA copy number in AD patients versus MCI subjects was documented in the Italian cohort, contrasting with the opposite trend observed in the Spanish cohort [48]. Mitochondrial quality control mechanisms, including mitophagy and mitochondrial dynamics, play a role in regulating mtDNA. Mitochondrial dynamics specifically influence the number of mtDNA copies, with mitochondrial fusion being essential for maintaining these copy levels [49,50]. Opa1-Exon4b has been found to bind specifically to the D-loop region of mtDNA accompanied by an increase in mtDNA transcription and subsequently mtDNA copy number [51]. The increase in mtDNA dynamics can be coupled with an increase in ROS production. Conversely, oxidative stress induces damage to mitochondrial DNA (mtDNA), and this compromised mtDNA further amplifies the production of reactive oxygen species (ROS), intensifying oxidative stress and perpetuating a self-reinforcing harmful cycle. Consequently, strategies aimed at reducing mtDNA levels under conditions of oxidative stress could serve as a promising approach to mitigate ROS production and, thereby, regulate oxidative stress effectively [52,53,54].

In HC patients, we observed a slight increase in D-loop methylation from T0 to T2, while mtDNA copy number showed a minor decrease, remaining substantially stable. Conversely, in patients exhibiting clinical progression toward AD, methylation trajectories were significantly lower, and mtDNA copy number was already reduced at T0, increasing at T1 and especially at T2, reinforcing the idea of a possible compensatory mechanism. Taken collectively, these data align with evidence indicating that mitochondrial activity varies markedly among controls, MCI, and AD, reflecting different metabolic demands or distinct phases of mitochondrial dysfunction. It can be postulated that the reduction in D-loop methylation observed in patients destined to develop AD represents a compensatory attempt in response to the emergent rise in mitochondrial activity or constitutes an early marker of nascent mitochondrial dysfunction characteristic of disease development. Supporting our analyses, other studies assessing gene expression in four cortical brain regions have demonstrated that genes associated with mitochondrial energy production were notably upregulated in MCI brains and, to a greater extent, in AD patients. This indicates that, in the brains of individuals with MCI, genes involved in energy metabolism are strongly upregulated, whereas in AD, these same genes are typically downregulated [55]. In a subsequent study, Lunnon et al. [56] observed reduced nuclear gene expression along with a concurrent increase in the expression of mitochondrial encoded OXPHOS genes in white blood cells of MCI and AD patients versus controls, with the differences being more pronounced in individuals with AD compared to controls. Similarly, Blanch et al. [25] observed increased expression of the mitochondrial gene *MT-ND1* (mitochondrially encoded NADH dehydrogenase 1) in patients at Braak stages V/VI, compared with both controls and patients at stages I/II. The authors suggested that the elevated MT-ND1 expression in advanced stages may result from the reduced MT-ND1 methylation levels detected during the early stages of the disease [25]. Based on this evidence and our results, we hypothesized that the decrease in D-loop methylation over time in patients, relative to controls, may result in an increase in mtDNA copy number as an effort to compensate for ongoing mitochondrial dysfunction. However, the increase in mtDNA copy number could lead to elevated expression of mitochondrial genes, driving increased ROS production, cell death, and progressive cognitive deficits, including memory impairment. Although a high mtDNA copy number may initially mitigate the risk of complete loss of mitochondrial function, it does not eliminate damage. In the long term, mitochondria inevitably become impaired: the continuous accumulation of compromised mtDNA hinders ATP production, and oxidative damage further exacerbates the accumulation of pathological proteins such as beta-amyloid and tau [57,58]. Compensatory increases in mtDNA copy number may temporarily support cellular bioenergetics, but ultimately, they may prove ineffective or even deleterious, amplifying oxidative stress and contributing to pathological advancement [47,57,59]. Furthermore, aging processes may contribute to the accumulation of mtDNA mutations which progressively lead to ROS production and bioenergetics defects [47,60,61].

### 3.3. Linking Cognitive Performance to Mitochondrial Dynamics: Dual Molecular Indicators for Alzheimer’s Progression

In our study, we assessed the association among MMSE scores, D-loop methylation, and mtDNA copy number, showing that in advanced stages (T2), cognitive decline is associated with a significant reduction in methylation and a compensatory increase in mtDNA copy number. These findings suggest that progressive loss of DNA methylation may reflect early epigenetic dysregulation associated with cognitive decline and may contribute to the molecular mechanisms underlying AD progression. Conversely, the increase in mtDNA copy number may represent a compensatory mitochondrial response to accumulating cellular stress or early neurodegenerative processes. Overall, the observed association supports the hypothesis that alterations in mitochondrial dynamics accompany cognitive decline and may serve as early molecular indicators of progression toward AD. Taken together, the findings indicate that cognitive decline, as assessed by the MMSE, is accompanied by measurable biological alterations at both epigenetic and mitochondrial levels. The combined assessment of D-loop methylation and mtDNA copy number may thus provide early molecular indicators useful for identifying individuals at increased risk of progressing toward AD.

### 3.4. Limitation of the Study

Our subgroup analyses, particularly those stratified by gender and APOE ε4 carrier status, were conducted on relatively small sample sizes, as these represented the largest samples available and the maximum number of participants that could be extracted from our cohort. This reduction in group size limits the statistical power of these specific comparisons, increasing the risk of Type II errors and potentially affecting the generalizability of our conclusions. Consequently, these findings should be interpreted with caution and are intended to be exploratory and hypothesis-generating, requiring confirmation in larger, independent cohorts.

Lastly, this study focused exclusively on mtDNA metrics. The correlation between mitochondrial epigenetic changes and established AD fluid biomarkers such as Aβ and p-Tau remains to be elucidated. Integrating these distinct molecular signatures will be a key objective of our future research projects, aiming to provide a more comprehensive understanding of the interplay between mitochondrial dysfunction and AD-related proteinopathy.

Given these factors, including the limited but maximally available sample size from our cohort, our results should be viewed as preliminary signals of systemic alterations rather than definitive diagnostic biomarkers.

## 4. Materials and Methods

### 4.1. Study Population and Design

**Figure 5:**
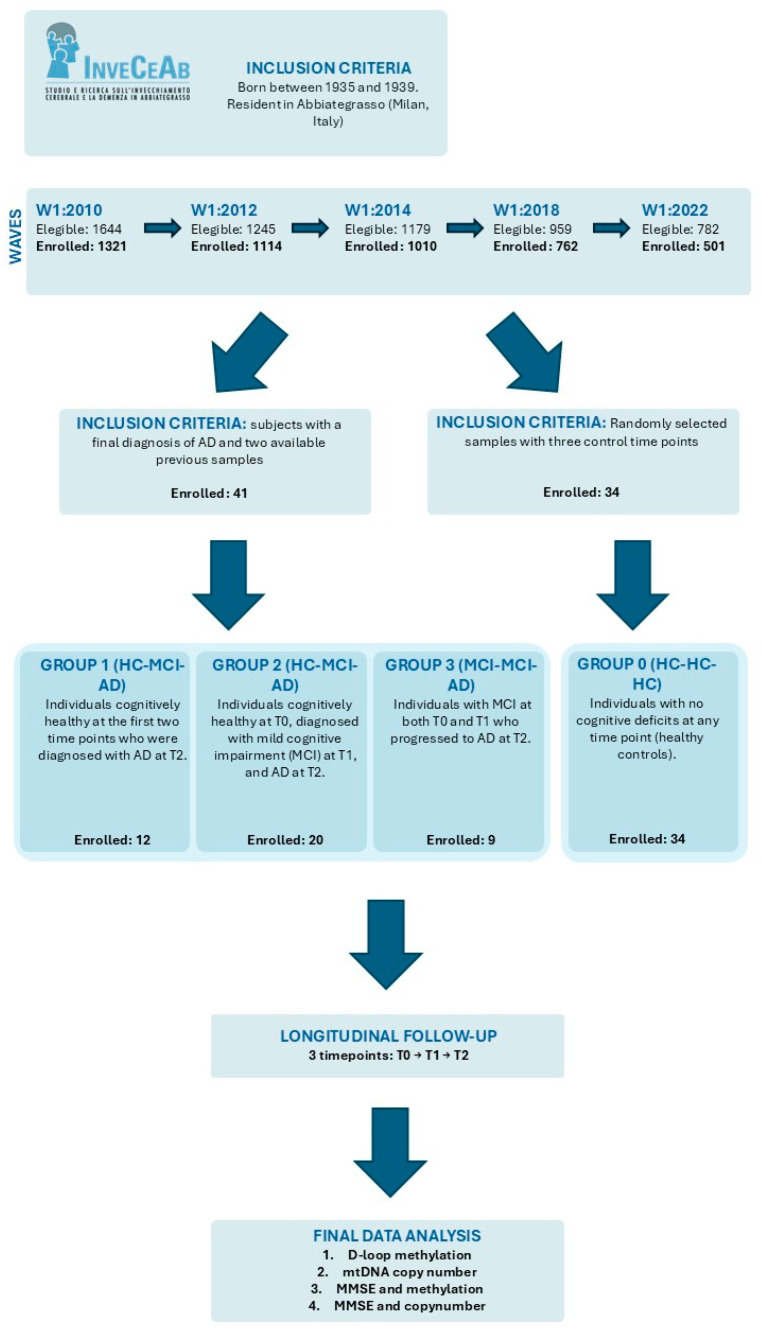
Flow diagram illustrating the InveCe.Ab study waves, participant selection, and measures of interest in the present study. The upper panel describes the inclusion criteria for the population-based InveCe.Ab cohort. The central panel shows the flow of participants across the five study waves. The panel reports the participants included in the analyses and their allocation into groups according to clinical progression. The final box summarizes the analyses performed on the study samples.

This study was designed as a retrospective longitudinal cohort study. The participants included in this study were recruited from the InveCe.Ab, whose method is detailed elsewhere [62]. Briefly, InveCe.Ab (Invecchiamento Cerebrale ad Abbiategrasso) is a longitudinal, population-based study on brain aging, involving the assessment and long-term monitoring of the physical conditions and cognitive status. The target population comprises all the Abbiategrasso (a city near Milan, Italy) residents born between 1935 and 1939 and a reference sample, according to Population Register data, of 1773 people. Among this, 1321 subjects decide to participate in the study [62]. Participants undergo repeated in-person assessments every 2 years for two follow-up waves (years 2010, 2012, and 2014) and every 4 years thereafter (year 2018 and 2022). At baseline and follow-ups, data were collected through clinical examinations, neurpsychological battery, biochemical tests, and structured interviews by trained personnel (geriatrician, neuropsychologists, biologists, interviewers). Written informed consent was obtained from all individuals prior to participation. The study was conducted in accordance with the ethical standards of the Declaration of Helsinki and received approval from the Ethics Committee of the University of Pavia (approval date: 6 October 2009; protocol no. 3/2009). In this study a total of 75 individuals wihout dementia were selected based on their cognitive trajectory at three time points: baseline, a 4-year follow-up, and a final evaluation after another 4 years. Participants were stratified into four groups: Group 0 (HC-HC-HC, n = 34) = Patients with no cognitive deficits at any time point (healthy controls); Group 1 (HC-HC-AD, n = 12) = Patients cognitively healthy at two time points, diagnosed with AD at T2; Group 2 (HC-MCI-AD, n = 20) = Patients cognitively healthy at T0, with mild cognitive impairment (MCI) at T1, and AD at T2; and Group 3 (MCI-MCI-AD, n = 9) = Patients with MCI at both T0 and T1, and AD at T2 (Table 9). Only subjects with available biological samples at all three time points were included in the present longitudinal analyses. Figure 5 shows the study design and measurements used for the present study.

### 4.2. Multidimensional Assessment

A comprehensive multidimensional evaluation was conducted. A geriatrician obtained an extensive and structured medical history, supported by available clinical records, documented ongoing pharmacological treatments, reviewed laboratory findings, and carried out a physical examination with particular focus on neurological signs and symptoms.

Neuropsychological assessment was performed by trained neuropsychologists and included an extensive cognitive test battery. Global cognitive functioning was assessed using MMSE. Specific cognitive domains were evaluated as follows: language through category fluency tasks; memory using the Rey Auditory Verbal Learning Test; attention via attentional matrices; executive functioning with the Trail Making Test and Raven’s Colored Progressive Matrices; and visuospatial abilities and constructional praxis through the Clock Drawing Test and the Rey–Osterrieth Complex Figure Test. Test scores were adjusted for age and education in accordance with Italian normative data, and cognitive impairment was defined based on established cutoff values. Additional details regarding the assessment tools are provided in the study protocol [62].

Following clinical and neuropsychological evaluations, the physician and the neuropsychologist independently formulated preliminary diagnostic hypotheses within predefined categories: dementia, according to DSM-5 criteria [63] and mild cognitive impairment, based on Petersen’s criteria [64]. The etiological diagnosis of probable Alzheimer’s disease was based on the presence of a typical amnestic cognitive profile and clinical course, in the absence of alternative neurological or psychiatric conditions. Individuals showing no evidence of cognitive deficits and without major psychiatric conditions, such as clinically significant depression or psychosis, were classified as cognitively normal.

Upon completion of the multidimensional assessment, all available clinical and neuropsychological data were jointly reviewed by an expert geriatrician and a clinical neuropsychologist, who reached a consensus final diagnosis within the aforementioned diagnostic categories. Neuroimaging or cerebrospinal fluid/plasma biomarker data were not available for this population-based cohort. Therefore, diagnoses were established on the basis of a comprehensive clinical and neuropsychological evaluation, in accordance with internationally accepted clinical diagnostic criteria

### 4.3. DNA Isolation from Blood Samples

All samples were collected and processed simultaneously. DNA extraction, bisulfite conversion, pyrosequencing, and qPCR were performed concurrently, with technical replicates included to assess assay precision. Conducting all experiments at the same time minimized potential batch effects, ensuring that observed longitudinal changes reflect true biological variation.

Total genomic DNA was extracted from whole blood using the Maxwell^®^ CSC 48 instrument (Promega, Madison, WI, USA) and the Maxwell^®^ CSC Blood DNA Kit (Promega, Madison, WI, USA), following the manufacturer’s instructions. DNA quantification was performed using a NanoDrop™ One/OneC UV-Vis microvolume spectrophotometer (Thermo Fisher Scientific, Waltham, MA, USA), which quantifies DNA, RNA, and proteins using only 1–2 µL of sample. The instrument also detects contaminants, providing accurate concentration measurements.

### 4.4. mtDNA Copy Number Quantification

mtDNA copy number was determined via quantitative PCR using 10 ng of total cellular DNA as input. Primers targeting the D-loop regulatory region of mtDNA (chrM: 3313–3322) [29,30] and *GAPDH* as a nuclear housekeeping gene were used (Table 10). Each qPCR reaction (15 µL) contained 200 nM of each oligonucleotide (Metabion, Planneg, Germany), 7.5 μL of SYBR Green SuperMix (BioRad, Hercules, CA, USA), 5.9 μL of Nuclease-free H_2_O, and 1 μL of DNA template (10 ng/μL) or water control.

Cycle threshold (Ct) values were recorded automatically. Relative mtDNA content was calculated by comparing the mean Ct of *mtDNA (D-loop)* and *GAPDH* using the following equations [65]:

ΔCt = Ct_nDNA − Ct_mtDNA(1)

Relative mtDNA content = 2 × 2^ΔCt^ (2)

### 4.5. D-Loop Methylation Analysis

For each sample, 500 ng of DNA was treated with sodium bisulfite using the EpiTect Fast Bisulfite Kit (Qiagen, Hilden, Germany), converting unmethylated cytosines to uracil. DNA methylation was analyzed via pyrosequencing on the heavy (H)-strand of the mtDNA D-loop region (nt 16,417 to 73, GenBank: J01415.2), covering three CpG sites.

A 226 bp amplicon was generated using previously published primers [29]. PCR was performed in a 25 μL reaction containing 12 μL of PyroMark PCR Master Mix 2×, 2.5 μL of CoralLoad Concentrate 10×, 2 μL of forward and primer mix (2.5 μM), RNase-free water (variable), and 10 ng of bisulfite converted DNA (Qiagen, Hilden, Germany). The following procedure was used for the PCR amplifications: 15 min at 95 °C, then 45 cycles of 30 s each at 94 °C, 56 °C, and 72 °C, with a final extension of 10 min at 72 °C. PCR products were purified and sequenced using the PyroMark Q48 Autoprep. The PyroMark Q48 Adv. CpG reagents (Qiagen, Hilden Germany), PyroMark Q48 Autoprep 4.2.1 software, and the manufacturer’s suggested protocols were used to determine the levels of DNA methylation. Methylation levels were calculated as the percentage of 5-methylcytosine at the three CpG sites within the amplicon.

### 4.6. Genotype Stratification

Genomic DNA was extracted from blood samples using the Maxwell^®^ 16 system (Promega Corporation, Madison, WI, USA). Genotyping analysis was conducted by real-time polymerase chain reaction allelic discrimination using TaqMan^®^ probes in a CFX 384 real-time PCR system (Bio-Rad, Hercules, CA, USA) and available pre-made assays (Applied Biosystems, Foster City, CA, USA). The TOMM40 rs2075650 was analyzed using the inventoried assay C_3084828. Allele calling analyzed by Real-Time PCR was based on the clustering algorithm implemented in CFX Manager™ software, version 3.1 (Bio-Rad, Hercules, CA, USA). APOE common variants (ε2, ε3, ε4) were determined from the combination of two SNPs, rs7412 and rs429358, within codons 112 and 158 of the APOE gene, respectively (C_904973 and C_3084793 assays for rs7412 and rs429358, respectively) [66].

### 4.7. Statistical Analysis

Statistical analyses were performed in R (version 4.5.0). Baseline demographic and clinical and molecular characteristics were summarized using appropriate descriptive statistics; categorical variables were expressed as counts and percentages; and continuous variables as means and standard deviations or medians and interquartile ranges according to their distribution. Normality was assessed using the Shapiro–Wilk test and inspection of Q–Q plots, and the homogeneity of variances across diagnostic groups was evaluated with Bartlett’s test. Given the known influence of blood cell composition on mitochondrial measures, white blood cell and platelet counts were examined as potential confounders. Differences between groups across time points were tested, and repeated-measures correlation analyses were conducted to assess their relationship with D-loop methylation and mtDNA copy number over time. Longitudinal changes in methylation levels and copy number were modeled using linear mixed-effects models with a random intercept for the subject to account for within-individual correlation. Fixed effects included time, diagnostic group, and their interaction, with additional adjustment for age and sex; APOE and TOMM40 genotypes were included as dichotomous variables, defined, respectively, as ε4 carrier status (presence vs. absence of at least one ε4 allele) and TOMM40 G allele carrier status (presence vs. absence of G), and their interaction was also entered. Model assumptions were checked by visual inspection of residuals and Q–Q plots and by Shapiro–Wilk tests on residuals; 95% confidence intervals were obtained using Wald methods, and, where indicated, robust variance–covariance estimators (CR2) were applied. To disentangle between- and within-subject effects in the associations among methylation, copy number, and global cognition (MMSE), individual baseline values (e.g., *MMSE baseline*) were used to capture stable between-subject differences, whereas deviations from baseline over time (e.g., *MMSE longitudinal*) represented within-subject change. The impact of cognitive performance on methylation and copy number, and of methylation on copy number, was then examined in mixed-effects models including both baseline and longitudinal components of MMSE or methylation and their interactions with time. Estimated marginal means and pairwise comparisons between groups and time points were derived from the fitted models using Tukey adjustment for multiple testing. All tests were two-sided, and a *p* value < 0.05 was considered statistically significant. No data imputation was performed, as longitudinal molecular data were complete for all included subjects across the selected time points.

## 5. Conclusions

Our findings indicate a potential association between D-loop mitochondrial methylation and different AD stages. While these changes are detectable in peripheral blood, they should be interpreted with caution as they might be influenced by systemic factors; nonetheless, they offer an interesting starting point for investigating D-loop methylation as a potential surrogate indicator of disease-related shifts. Epigenetic biomarkers have garnered substantial interest as valuable tools for diagnosis, prognosis, and therapeutic monitoring across various conditions [67]. In the context of AD, multiple investigations have reported peripheral DNA methylation alterations in several distinct genes [68,69,70].

While we acknowledge the limitation of a restricted number of individuals, statistical analysis confirmed that the study was adequate to detect differences in D-loop methylation, even those small or robust, among the investigated groups. The key strength of our study lies in its longitudinal dataset, which allows us to track varying patterns of pathological progression that align with findings previously reported in animal studies.

The increasing trend in methylation levels observed in cognitively healthy controls and the sharp decline coinciding with clinical diagnosis in patients reinforce the conceptual link between mitochondrial methylation and cognitive health. Significant differences identified across groups and time points further support this interpretation. Notably, the decrease in D-loop methylation in peripheral blood either precedes or coincides with clinical diagnosis. This timing could establish mitochondrial methylation as an early indicator, potentially useful for identifying at-risk subjects or those in the preclinical phase.

Supporting the relevance of mitochondrial modifications in AD progression, our data also shows that mtDNA copy number was significantly reduced at T0 in patients who later developed AD compared with subjects who remained cognitively healthy. Over time, while copy number remained relatively stable in controls, it showed an upward tendency in subjects progressing to AD. This increase suggests a potential compensatory mechanism aimed at counteracting the increasing mitochondrial dysfunction. Simultaneously, this phenomenon provides a possible therapeutic target aimed at reducing the mtDNA copy number and, consequently, reducing the state of oxidative stress, which is one of the main characteristics of the disease. The strength of our conclusions is reinforced by the demonstrated correlation of D-loop and mtDNA with the MMSE, in the same direction.

Overall, the findings provide preliminary evidence that D-loop methylation and variations in mtDNA copy number could be relevant peripheral features related to AD progression. Future extensive investigations involving larger cohorts will be crucial to confirm and expand these findings, as well as to unravel the precise causal role of mitoepigenetic modifications and mtDNA dynamics in AD pathogenesis.

## Figures and Tables

**Table 1:** Results of the linear mixed-effects model examining longitudinal changes in D-loop methylation across diagnostic groups and time points (T0, T1, and T2). Summary of study cohort: Group 0 (HC-HC-HC, n = 34) = Patients with no cognitive deficits at any time point (healthy controls); Group 1 (HC-HC-AD, n = 12) = Patients cognitively healthy at two time points, diagnosed with AD at T2; Group 2 (HC-MCI-AD, n = 20) = Patients cognitively healthy at T0, with MCI at T1, and AD at T2; Group 3 (MCI-MCI-AD, n = 9) = Patients with MCI at both T0 and T1, and AD at T2.

Parameters	Beta	SE	95% Confidence Interval	*p*-Value
(Intercept)	1.78	1.27	[−0.72–4.28]	0.164
T1	−0.02	0.15	[−0.31–0.27]	0.880
T2	0.30	0.19	[−0.07–0.67]	0.109
Group 1	0.02	0.18	[−0.34–0.39]	0.892
Group 2	0.45	0.15	[0.14–0.75]	0.004
Group 3	−0.17	0.20	[−0.57–0.23]	0.410
Age	0.02	0.02	[−0.01–0.06]	0.175
Sex (M)	−0.15	0.08	[−0.30–0.00]	0.056
APOE ε4+ carrier	0.02	0.17	[−0.32–0.36]	0.899
TOMM40(G)+ carrier	−0.05	0.14	[−0.33–0.22]	0.698
T1: Group 1	−0.01	0.26	[−0.52–0.50]	0.972
T2: Group 1	−0.86	0.26	[−1.37–−0.35]	0.001
T1: Group 2	−0.29	0.22	[−0.71–0.14]	0.188
T2: Group 2	−1.17	0.22	[−1.60–−0.75]	<0.001
T1: Group 3	−0.54	0.29	[−1.10–0.03]	0.064
T2: Group 3	0.00	0.29	[−0.56–0.57]	0.994
APOE ε4+/TOMM40(G) + carrier	0.04	0.23	[−0.41–0.49]	0.860

**Table 2:** Pairwise group comparisons post hoc analysis for D-loop methylation level between the selected groups at T0, T1 after four years, and T2 after another for years. Summary of study cohort: Group 0 (HC-HC-HC, n = 34) (green) = Patients with no cognitive deficits at any time point (healthy controls); Group 1 (HC-HC-AD, n = 12) (orange) = Patients cognitively healthy at two time points, diagnosed with AD at T2; Group 2 (HC-MCI-AD, n = 20) (blue) = Patients cognitively healthy at T0, with MCI at T1, and AD at T2; Group 3 (MCI-MCI-AD, n = 9) (fuchsia) = Patients with MCI at both T0 and T1, and AD at T2.

Pairwise Group Comparisons Post Hoc Analysis				
Contrast	Mean Difference	SE	95% Confidence Interval	*p*-Value
T0—Group 0 VS. Group 1	−0.02	0.18	[−0.50–0.45]	0.999
T0—Group 0 VS. Group 2	−0.45	0.15	[−0.85–−0.046]	0.022
T0—Group 0 VS. Group 3	0.17	0.20	[−0.36–0.70]	0.842
T0—Group 1 VS. Group 2	−0.42	0.20	[−0.93–0.09]	0.153
T0—Group 1 VS. Group 3	0.19	0.24	[−0.43–0.82]	0.851
T0—Group 2 VS. Group 3	0.61	0.22	[0.04–1.19]	0.029
T1—Group 0 VS. Group 1	−0.01	0.18	[−0.49–0.46]	0.100
T1—Group 0 VS. Group 2	−0.16	0.15	[−0.56–0.24]	0.728
T1—Group 0 VS. Group 3	0.70	0.20	[0.17–1.24]	0.004
T1—Group 1 VS. Group 2	−0.14	0.20	[−0.66–0.37]	0.888
T1—Group 1 VS. Group 3	0.72	0.24	[0.09–1.35]	0.016
T1—Group 2 VS. Group 3	0.87	0.22	[0.29–1.44]	0.001
T2—Group 0 VS. Group 1	0.83	0.18	[0.35–1.31]	<0.001
T2—Group 0 VS. Group 2	0.73	0.15	[0.33–1.13]	<0.001
T2—Group 0 VS. Group 3	0.17	0.20	[−0.36–0.70]	0.848
T2—Group 1 VS. Group 2	−0.10	0.20	[−0.62–0.41]	0.954
T2—Group 1 VS. Group 3	−0.67	0.24	[−1.29–−0.04]	0.031
T2—Group 2 VS. Group 3	−0.56	0.22	[−1.13–0.01]	0.056

**Table 3:** Within-group time comparisons post hoc analysis for D-loop methylation level between the selected groups at T0, T1 after four years, and T2 after another four years. Summary of study cohort: Group 0 (HC-HC-HC, n = 34) (green) = Patients with no cognitive deficits at any time point (healthy controls); Group 1 (HC-HC-AD, n = 12) (orange) = Patients cognitively healthy at two time points, diagnosed with AD at T2; Group 2 (HC-MCI-AD, n = 20) (blue) = Patients cognitively healthy at T0, with MCI at T1, and AD at T2; Group 3 (MCI-MCI-AD, n = 9) (fuchsia) = Patients with MCI at both T0 and T1, and AD at T2.

Within-Group Time Comparisons Post Hoc Analysis				
Contrast	Mean Difference	SE	95% Confidence Interval	*p*-Value
Group 0 T0–T1	0.02	0.148	[−0.33–0.37]	0.988
Group 0 T0–T2	−0.30	0.189	[−0.75–0.14]	0.245
Group 0 T1–T2	−0.33	0.148	[−0.68–0.02]	0.073
Group 1 T0–T1	0.03	0.232	[−0.52–0.58]	0.990
Group 1 T0–T2	0.55	0.260	[−0.06–1.17]	0.085
Group 1 T1–T2	0.52	0.232	[−0.03–1.07]	0.065
Group 2 T0–T1	0.31	0.185	[−0.13–0.75]	0.219
Group 2 T0–T2	0.87	0.219	[0.36–1.39]	<0.001
Group 2 T1–T2	0.56	0.185	[0.13–1.00]	0.007
Group 3 T0–T1	0.56	0.265	[−0.07–1.19]	0.091
Group 3 T0–T2	−0.31	0.290	[−0.99–0.38]	0.542
Group 3 T1–T2	−0.87	0.265	[−1.49–−0.24]	0.004

**Table 4:** Results of the linear mixed-effects model examining longitudinal changes in mtDNA copy number across diagnostic groups and time points (T0, T1, and T2). Summary of study cohort: Group 0 (HC-HC-HC, n = 34) (green) = Patients with no cognitive deficits at any time point (healthy controls); Group 1 (HC-HC-AD, n = 12) (orange) = Patients cognitively healthy at two time points, diagnosed with AD at T2; Group 2 (HC-MCI-AD, n = 20) (blue) = Patients cognitively healthy at T0, with MCI at T1, and AD at T2; Group 3 (MCI-MCI-AD, n = 9) (fuchsia) = Patients with MCI at both T0 and T1, and AD at T2.

Parameters	Beta	SE	95% Confidence Interval	*p*-Value
(Intercept)	181.33	55.66	[+72.25–+290.43]	0.002
T1	−7.95	6.27	[−20.25–+4.34]	0.206
T2	−2.16	8.10	[−18.03–+13.71]	0.790
Group 1	−37.14	7.86	[−52.55–−21.73]	<0.001
Group 2	−23.59	6.59	[−36.51–−10.67]	<0.001
Group 3	−20.02	8.74	[−37.16–−2.89]	0.023
Age	−1.34	0.74	[−2.79–+0.11]	0.075
Sex (M)	−0.36	3.40	[−7.03–+6.29]	0.915
APOE ε4 + carrier	3.70	7.50	[−11.00–+18.40]	0.623
TOMM40(G) + carrier	−1.29	6.09	[−13.22–+10.64]	0.834
T1: Group 1	30.08	10.83	[+8.86–+51.31]	0.006
T2: Group 1	45.85	10.83	[+24.63–+67.08]	<0.001
T1: Group 2	16.17	9.09	[−1.64–+33.98]	0.077
T2: Group 2	46.25	9.09	[+28.43–+64.06]	<0.001
T1: Group 3	23.39	12.09	[−0.31–+47.08]	0.055
T2: Group 3	20.26	12.09	[−3.43–+43.96]	0.096
APOE ε4+/TOMM40(G) + carrier	−3.19	10.10	[−22.99–+16.62]	0.753

**Table 5:** Pairwise group comparisons post hoc analysis for mtDNA copy numbers between the selected groups at T0, T1 after four years, and T2 after another four years. Summary of study cohort: Group 0 (HC-HC-HC, n = 34) (green) = Patients with no cognitive deficits at any time point (healthy controls); Group 1 (HC-HC-AD, n = 12) (orange) = Patients cognitively healthy at two time points, diagnosed with AD at T2; Group 2 (HC-MCI-AD, n = 20) (blue) = Patients cognitively healthy at T0, with MCI at T1, and AD at T2; Group 3 (MCI-MCI-AD, n = 9) (fuchsia) = Patients with MCI at both T0 and T1, and AD at T2.

Pairwise Group Comparisons Post Hoc Analysis				
Contrast	Mean Difference	SE	95% Confidence Interval	*p*-Value
T0—Group 0 VS. Group 1	37.14	7.86	[0.36–0.93]	<0.001
T0—Group 0 VS. Group 2	23.59	6.59	[0.11–0.59]	0.002
T0—Group 0 VS. Group 3	20.02	8.74	[−0.01–0.63]	0.104
T0—Group 1 VS. Group 2	−13.55	8.50	[−0.60–0.02]	0.384
T0—Group 1 VS. Group 3	−17.12	10.30	[−0.71–0.04]	0.344
T0—Group 2 VS. Group 3	−3.56	9.41	[−0.39–0.30]	0.981
T1—Group 0 VS. Group 1	7.06	7.86	[−0.24–0.34]	0.806
T1—Group 0 VS. Group 2	7.42	6.59	[−0.17–0.31]	0.674
T1—Group 0 VS. Group 3	−3.36	8.74	[−0.43–0.20]	0.981
T1—Group 1 VS. Group 2	0.36	8.50	[−0.29–0.33]	1.000
T1—Group 1 VS. Group 3	−10.42	10.30	[−0.54–0.21]	0.741
T1—Group 2 VS. Group 3	−10.78	9.41	[−0.53–0.15]	0.661
T2—Group 0 VS. Group 1	−8.71	7.86	[−0.39–0.18]	0.685
T2—Group 0 VS. Group 2	−22.66	6.59	[−0.45–0.03]	0.004
T2—Group 0 VS. Group 3	−0.24	8.74	[−0.34–0.30]	1.000
T2—Group 1 VS. Group 2	−13.95	8.50	[−0.41–0.20]	0.358
T2—Group 1 VS. Group 3	8.47	10.30	[−0.29–0.46]	0.843
T2—Group 2 VS. Group 3	22.42	9.41	[−0.15–0.53]	0.084

**Table 6:** Within-group time comparisons post hoc analysis for mtDNA copy number between the selected groups at T0, T1 after four years, and T2 after another for years. Summary of study cohort: Group 0 (HC-HC-HC, n = 34) (green) = Patients with no cognitive deficits at any time point (healthy controls); Group 1 (HC-HC-AD, n = 12) (orange) = Patients cognitively healthy at two time points, diagnosed with AD at T2; Group 2 (HC-MCI-AD, n = 20) (blue) = Patients cognitively healthy at T0, with MCI at T1, and AD at T2; Group 3 (MCI-MCI-AD, n = 9) (fuchsia) = Patients with MCI at both T0 and T1, and AD at T2.

Within-Group Time Comparisons Post Hoc Analysis				
Contrast	Mean Difference	SE	95% Confidence Interval	*p*-Value
Group 0 T0–T1	7.95	6.27	[−0.05–0.37]	0.415
Group 0 T0–T2	2.16	8.10	[−0.24–0.29]	0.962
Group 0 T1–T2	−5.80	6.27	[−0.35–0.07]	0.626
Group 1 T0–T1	−22.13	9.77	[−0.76–−0.11]	0.064
Group 1 T0–T2	−43.69	11.00	[−1.09–−0.36]	<0.001
Group 1 T1–T2	−21.57	9.77	[−0.62–0.04]	0.073
Group 2 T0–T1	−8.22	7.79	[−0.38–0.14]	0.544
Group 2 T0–T2	−44.09	9.33	[−0.85–−0.23]	<0.001
Group 2 T1–T2	−35.87	7.79	[−0.68–−0.16]	<0.001
Group 3 T0–T1	−15.43	11.10	[−0.64–0.11]	0.352
Group 3 T0–T2	−18.11	12.30	[−0.71–0.11]	0.305
Group 3 T1–T2	−2.67	11.10	[−0.41–0.34]	0.969

**Table 7:** Results of the linear mixed-effects model examining longitudinal changes in MMSE and D-loop methylation across time points (T0, T1, and T2).

Parameters	Beta	SE	95% Confidence Interval	*p*-Value
**(Intercept)**	1.91	1.67	[−1.36–+5.18]	0.255
**T1**	−0.31	1.22	[−2.71–+2.08]	0.797
**T2**	1.35	1.29	[−1.17–+3.88]	0.294
**Age**	0.02	0.02	[−0.02–+0.05]	0.342
**Sex (M)**	−0.18	0.08	[−0.35–−0.01]	0.037
**APOE ε4 + carrier**	0.08	0.19	[−0.29–+0.45]	0.662
**TOMM40(G) + carrier**	−0.08	0.15	[−0.38–+0.22]	0.600
**MMSE_baseline**	0.01	0.03	[−0.05–+0.07]	0.654
**APOE ε4+/TOMM40(G)+ carrier**	−0.01	0.25	[−0.51–+0.48]	0.952
**T1: MMSE_baseline**	0.01	0.04	[−0.08–+0.09]	0.903
**T2: MMSE_baseline**	−0.04	0.04	[−0.13–+0.05]	0.345
**T1: MMSE_longitudinal**	−0.02	0.03	[−0.07–+0.04]	0.502
**T2: MMSE_longitudinal**	0.05	0.01	[+0.02–+0.08]	<0.001

**Table 8:** LMM analysis comparisons for MMSE and mtDNA copy numbers between the selected groups at T0, T1 after four years, and T2 after another for years.

Parameters	Beta	SE	95% Confidence Interval	*p*-Value
**(Intercept)**	101.60	67.87	[−31.43–+234.63]	0.138
**T1**	−0.40	47.89	[−94.28–+93.47]	0.993
**T2**	−48.89	50.58	[−148.03–+50.24]	0.335
**Age**	−0.83	0.77	[−2.33–+0.67]	0.284
**Sex (M)**	2.53	3.51	[−4.35–+9.43]	0.473
**APOE ε4 + carrier**	1.95	7.72	[−13.19–+17.09]	0.802
**TOMM40(G) + carrier**	−5.87	6.20	[−18.03–+6.28]	0.347
**MMSE_baseline**	0.96	1.23	[−1.46–+3.38]	0.438
**APOE ε4+/TOMM40(G) + carrier**	−3.76	10.40	[−24.13–+16.62]	0.719
**T1: MMSE_baseline**	0.10	1.70	[−3.23–+3.43]	0.952
**T2: MMSE_baseline**	1.91	1.76	[−1.53–+5.35]	0.279
**T1: MMSE_longitudinal**	0.43	1.08	[−1.69–+2.56]	0.691
**T2: MMSE_longitudinal**	−2.06	0.59	[−3.22–−0.90]	<0.001

**Table 9:** Summary of study cohort: Group 0 (HC-HC-HC, n = 34) = Patients with no cognitive deficits at any time point (healthy controls); Group 1 (HC-HC-AD, n = 12) = Patients cognitively healthy at two time points, diagnosed with AD at T2; Group 2 (HC-MCI-AD, n = 20) = Patients cognitively healthy at T0, with MCI at T1, and AD at T2; Group 3 (MCI-MCI-AD, n = 9) = Patients with MCI at both T0 and T1, and AD at T2. SD = Standard Deviation.

Phenotype	Group 0 at t0	Group 1 at t0	Group 2 at t0	Group 3 at t0
**Blood Sample**	34	12	20	9
**Mean Age ±** **SD**	75.97 ± (2.08)	75.33 ± (2.39)	75.20 ± (2.91)	76.00 ± (2.00)
**Sex (F)**	16	8	14	3
**Sex (M)**	18	4	6	6
**MMSE ±** **SD**	28.91 ± (1.19)	28.00 ± (1.81)	27.40 ± (3.35)	26.11 ± (1.36)
**APOE 2//3**	2	1	2	1
**APOE 3//3**	26	8	12	5
**APOE 3//4**	6	3	6	2
**APOE 4//4**	0	0	0	1
**TOMM40 AA**	28	7	15	5
**TOMM40 AG**	6	5	5	3
**TOMM40 GG**	0	0	0	1

**Table 10:** Primer sequences for RT-qPCR.

Region	Primer Forward	Primer Reverse
***mt-DNA (D-loop)***	CACCCAAGAACAGGGTTTGT	TGGCCATGGGTATGTTGTTA
***GAPDH***	CTGAACGGGAAGCTCACTGG	GGCAGGTTTTTCTAGACGGC

## Data Availability

The datasets generated and/or analyzed during the current study are available in the ZENODO repository (https://zenodo.org/) (10.5281/zenodo.17953073). Accession date 15 December 2025.

## Abbreviations

The following abbreviations are used in this manuscript:

AD	Alzheimer’s disease
APOE	Apolipoprotein E
Aβ	β-amyloid
DEGs	Differentially expressed genes
NFTs	Neurofibrillary tangles
Ptau	Hyperphosphorylated tau
HC	Healthy controls
OXPHOS	Oxidative phosphorylation
ROS	Reactive oxygen species
mtDNA	Mitochondrial DNA
nDNA	Nuclear DNA
HBB	Hemoglobin
IGF2	Insulin growth factor 2
T0	Baseline (time point 0)
T1	Follow-up (time point 1)
T2	Final evaluation (time point 2)
MCI	Mild cognitive impairment
MMSE	Mini-Mental State Examination
CNS	Central nervous system
MT-ND1	Mitochondrially encoded NADH dehydrogenase 1
Ct	Cycle threshold
